# Cloning and functional characterization of the rabbit C-C chemokine receptor 2

**DOI:** 10.1186/1471-2172-6-15

**Published:** 2005-07-07

**Authors:** Deshun Lu, Xiu-juan Yuan, Robert J Evans, Amy T Pappas, He Wang, Eric W Su, Chafiq Hamdouchi, Chandrasekar Venkataraman

**Affiliations:** 1 Divisions of Atherosclerosis, Cancer, Integrative Biology and Discovery Chemistry, Lilly Research Laboratories, Lilly Corporate Center, Indianapolis, Indiana 46285, USA

## Abstract

**Background:**

CC-family chemokine receptor 2 (CCR2) is implicated in the trafficking of blood-borne monocytes to sites of inflammation and is implicated in the pathogenesis of several inflammatory diseases such as rheumatoid arthritis, multiple sclerosis and atherosclerosis. The major challenge in the development of small molecule chemokine receptor antagonists is the lack of cross-species activity to the receptor in the preclinical species. Rabbit models have been widely used to study the role of various inflammatory molecules in the development of inflammatory processes. Therefore, in this study, we report the cloning and characterization of rabbit CCR2. Data regarding the activity of the CCR2 antagonist will provide valuable tools to perform toxicology and efficacy studies in the rabbit model.

**Results:**

Sequence alignment indicated that rabbit CCR2 shares 80 % identity to human CCR2b. Tissue distribution indicated that rabbit CCR2 is abundantly expressed in spleen and lung. Recombinant rabbit CCR2 expressed as stable transfectants in U-937 cells binds radiolabeled ^125^I-mouse JE (murine MCP-1) with a calculated *K*_*d *_of 0.1 nM. In competition binding assays, binding of radiolabeled mouse JE to rabbit CCR2 is differentially competed by human MCP-1, -2, -3 and -4, but not by RANTES, MIP-1α or MIP-1β. U-937/rabbit CCR2 stable transfectants undergo chemotaxis in response to both human MCP-1 and mouse JE with potencies comparable to those reported for human CCR2b. Finally, TAK-779, a dual CCR2/CCR5 antagonist effectively inhibits the binding of ^125^I-mouse JE (*IC*_50 _= 2.3 nM) to rabbit CCR2 and effectively blocks CCR2-mediated chemotaxis.

**Conclusion:**

In this study, we report the cloning of rabbit CCR2 and demonstrate that this receptor is a functional chemotactic receptor for MCP-1.

## Background

Chemokine receptors are seven-transmembrane G-protein coupled receptors that direct the migration of various immune cells to the sites of inflammation in addition to their pivotal role in maintaining immune cell homeostasis in various lymphoid compartments [1,2]. Chemokines (8–14 kDa molecular weight), the ligands of chemokine receptors, are broadly classified based on the positioning of the two conserved cysteines into the CC, CXC, C or CX_3_C family [3]. Binding of chemokines to their cognate receptors on immune cells triggers a signaling cascade primarily mediated by the G_αi _family of G proteins leading to a variety of cell effector functions including chemotaxis, degranulation, and release of pro-inflammatory cytokines such as TNF α [4].

The CC family chemokine receptor 2 (CCR2) is primarily expressed in almost all circulating monocytes. CCR2 is believed to mediate extravasation of blood monocytes to the sites of inflammation [5-7] and is implicated in the pathogenesis of several inflammatory diseases such as rheumatoid arthritis, multiple sclerosis and atherosclerosis [8,9]. MCP subfamily members, namely MCP-1, -2, -3, and -4, bind to CCR2 with high affinity [7,10,11]. Several studies involving genetic deletion of CCR2 or MCP-1, and antibody neutralization studies of MCP-1 have clearly established the critical role of the CCR2/MCP-1 axis in mediating several key pathogenic events in animal models of multiple sclerosis and atherosclerosis.

The major challenge in the development of small molecule chemokine receptor antagonists is the lack of cross-species activity to the receptor in the preclinical species. For example, BX-471, a small molecule antagonist for CCR1, exhibits low nano-molar affinity to the human receptor but has sub-micromolar to high micromolar affinity to the rat and mouse CCR1 receptors, respectively [12]. Importantly, BX-471 had intermediate affinity to the rabbit CCR1 receptor [13]. This imposes significant challenges to compound testing in efficacy and toxicology studies in the preclinical models.

In recent years, rabbits have been used to study the pathogenesis of inflammatory disease model. It has been recently shown that the therapeutic potential of thiazolidinediones in rabbit model of balloon injury and re-endothelialization is associated with a decrease in MCP-1 expression [14]. In addition, the efficacy of a CXCR2 antagonist has been successfully demonstrated in a chronic antigen induced arthritis model [15]. Moreover, CCR1 antagonists have therapeutic benefit in a rabbit allograft rejection model [13]. In the present study we report the cloning and functional characterization of the rabbit CCR2. We also demonstrate that the recently reported CCR2/CCR5 dual antagonist, TAK-779, exhibits high level of potency against rabbit CCR2 in both binding and chemotaxis assays.

## Results and Discussion

### Cloning and expression analysis of rabbit CCR2

**Figure 1:**
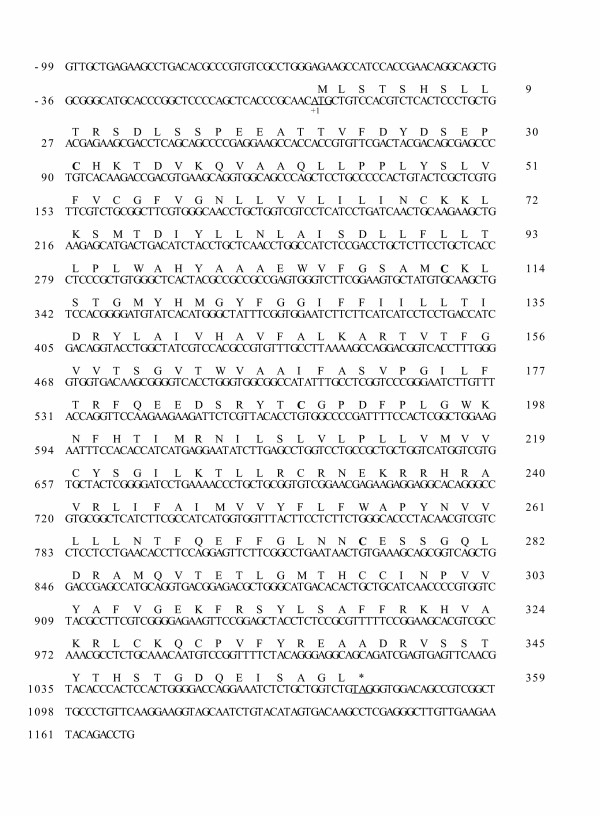
**Full-length rabbit CCR2 cDNA sequence**. The complete cDNA sequence is shown with nucleotides numbered from the putative initiation site (underlined). The deduced amino acid sequence is on the top of nucleotide sequence. Cysteines that form the disulfide bonds are labeled in bold for Cys-112, Cys-189 (SS model) and Cys-31, Cys-276 (2SS model), respectively.

**Figure 2:**
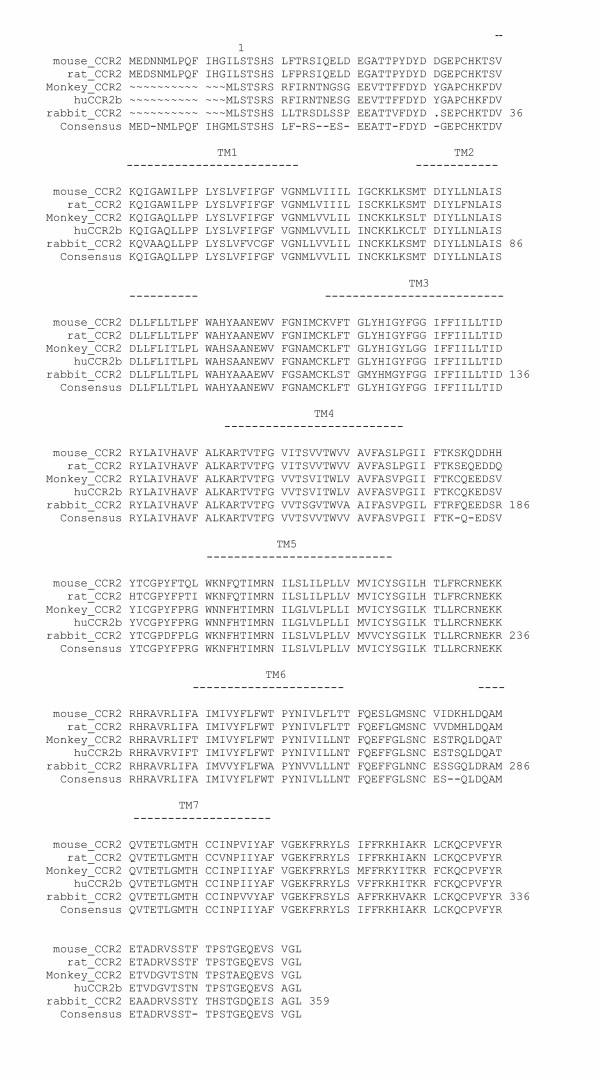
**CCR2 peptide sequence alignment**. The CCR2 peptide sequences from mouse, rat, monkey, human and rabbit are aligned and the consensus sequence is indicated in the bottom of the alignment. Transmembrane domains (TM) designated based on the human CCR2 sequence are also indicated.

**Figure 3:**
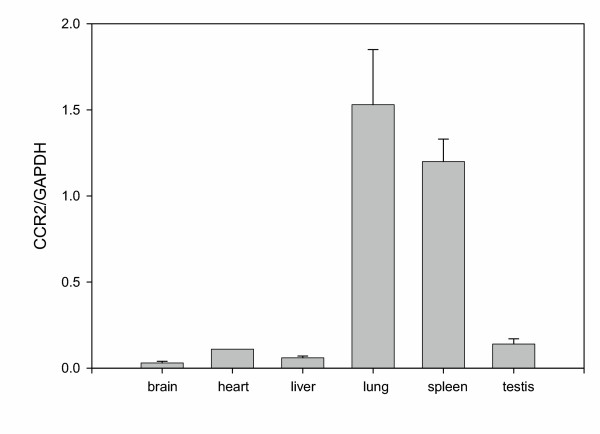
**Tissue distribution of rabbit CCR2**. The mRNA levels from rabbit tissues were quantitated by Taqman technique as described under "Materials and Methods". CCR2 mRNA levels are expressed as the ratio of CCR2 and GAPDH. Error bars represent the mean ± standard deviation of quadruplicate values.

Full-length cDNA sequence of rabbit CCR2 was cloned from total spleen RNA of New Zealand white rabbits using a PCR based method. The deduced amino acid sequence of rabbit CCR2 encoded a protein of 369 amino acids (Fig. 1) that shared 80 % identity to the human CCR2b receptor. The sequence alignment with the mouse, rat and rhesus monkey CCR2 is also depicted in Fig. 2. Rat and mouse CCR2 contained additional 13 amino acids at the N-terminus compared to human, monkey and rabbit CCR2. In addition, one amino acid deletion in rabbit CCR2 was observed at position 18 (Fig. 2). Cysteines that were essential for the disulfide bond formation were also conserved in rabbit CCR2. Based on the SS and 2SS model [16], cysteine pairs (Cys-72/Cys-119 and Cys-31/Cys-276) in rabbit CCR2 were highly conserved compared to its counterparts from all known species. Expression levels of rabbit CCR2 were also determined in various rabbit tissues using Taqman analysis. Rabbit CCR2 was abundantly expressed in lungs and spleen compared to low levels of expression in brain, heart, liver and testis (Fig. 3).

### Radioligand binding studies in rabbit CCR2 expressing cells

**Figure 4:**
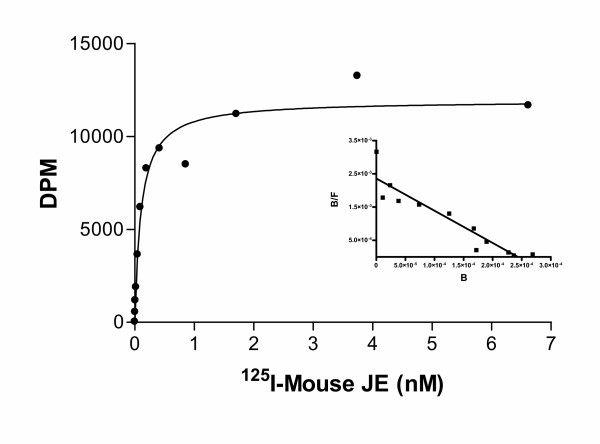
**Saturation binding of mouse JE to rabbit CCR2**. Various concentrations of ^125^I-mouse JE was added to U-937/rabbit CCR2 stable transfectants as described under "Materials and Methods". The nonspecific binding was determined by including 100-fold excess of unlabeled chemokine. Specific binding was calculated by subtracting nonspecific binding from total binding. The dissociation constant (*K*_*d*_) was calculated using the nonlinear curve fit. Each point represents mean values of the duplicates from one of two representative assays. The scatchard plot is depicted in the insert.

Stable cell lines expressing rabbit CCR2 in U-937 cells were generated to characterize the binding properties of radiolabeled human MCP-1 or mouse JE to rabbit CCR2. The binding affinity of radiolabeled human MCP-1 or mouse JE was measured using a competition binding assay of the respective cold ligands. As shown in Figs. 4, ^125^I-mouse JE showed high affinity with a calculated *K*_*d *_of 0.95 ± 0.02 nM and *B*_*max *_of 18829 ± 1555 dpm. In contrast, ^125^I-human MCP-1 has less binding affinity to rabbit CCR2 with lack of saturable binding as high as 6 nM of hot ligand (data not shown). Detailed examination of the binding characteristics of radiolabeled human MCP-1 to rabbit CCR2 will be necessary to resolve this discrepancy. To address the point, future studies are necessary to understand rabbit CCR2 binding characteristics and pharmacology with ^125^I-rabbit- MCP-1.

**Figure 5:**
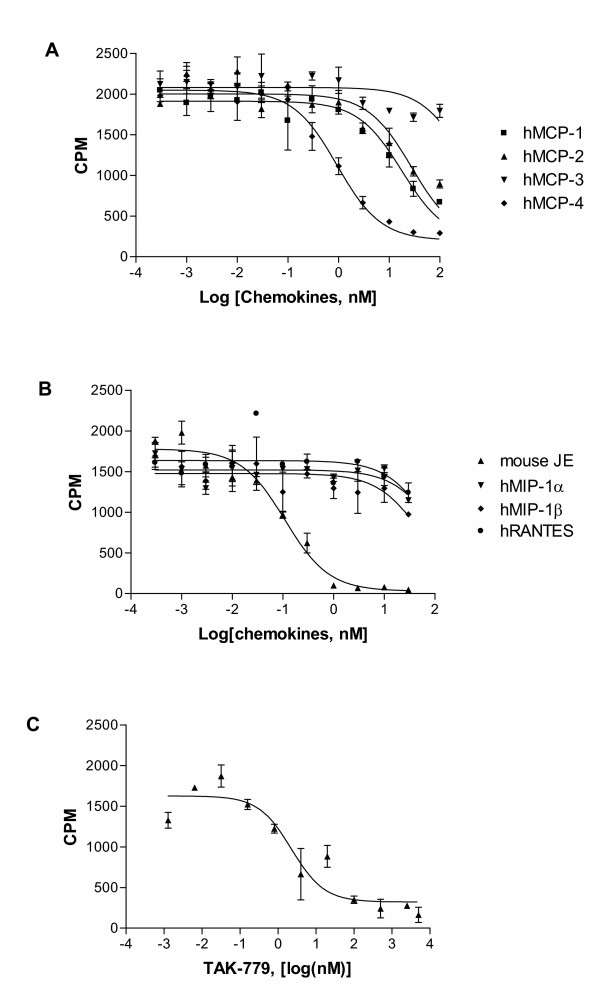
**Competition assays of various chemokines on U-937/rabbit CCR2 stable transfectants**. (A) U-937/rabbit CCR2 stable cells (104 cells/well) were incubated with ^125^I-mouse JE (0.11 nM) for 2 hours in the presence of unlabeled chemokines (Human MCP-1, -2, -3, -4) at concentrations as high as 30 nM. (B). U-937/rabbit CCR2 stable cells (10^4 ^cells/well) were incubated with ^125^I-mouse JE (0.11 nM) for 2 hours in the presence of unlabeled chemokines (mouse JE, hMIP-1α, hMIP-1β and hRANTES) at concentrations as high as 30 nM. (C) Competition binding assay was performed on U-937/rabbit CCR2 stable cells (10^4 ^cells/well) that were incubated with ^125^I-mouse JE (0.11 nM) for 2 hours in the presence of various concentrations of TAK-779. Each point represents mean values ± standard deviations of the duplicates from one of two representative assays.

Receptor pharmacology of rabbit CCR2 was studied using a variety of ligands that belonged to the MCP subfamily and irrelevant ligands such as RANTES, MIP-1α and MIP-1β. As expected, human MCP-1, -2, and -4 differentially competed the binding of radiolabeled mouse JE with *K*_*i *_values of 8.4, 13.5 and 0.44 nM, respectively. In contrast, cold MCP-3 failed to compete JE binding to rabbit CCR2 when used at concentrations as high as 30 nM (Fig. 5A). In addition, irrelevant ligands that bind with high affinity to CCR1 or CCR5 were unable to compete binding of radiolabeled mouse JE to rabbit CCR2, while cold JE competed binding with a *Ki *value of 0.116 nM (Fig. 5B). TAK-779 is a well characterized small molecule antagonist of both CCR2 and CCR5, and has a 27 nM affinity against human CCR2b receptor [17]. We tested the activity of TAK-779 on rabbit CCR2 transfected cells using radiolabeled mouse JE in a standard competition binding assay. As shown in Fig. 5C, TAK-779 was also highly potent against rabbit CCR2 with an *IC*_50 _of 2.3 nM.

### Functional characterization of Rabbit CCR2 stable cell line

**Figure 6:**
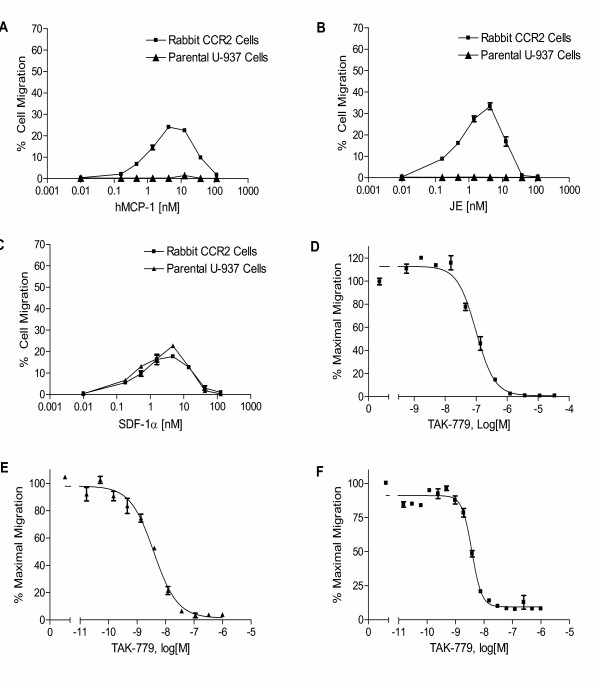
**Chemotaxis assay in U-937/rabbit CCR2 stable transfectants**. Chemotaxis assays were performed in parental U-937 or U-937/rabbit CCR2 stable transfectants using increasing concentrations of human MCP-1 (A), mouse JE (B) or human SDF-1α (C). The effect of TAK-779 on rabbit CCR2-induced chemotaxis was tested in the presence of 4 nM of mouse JE (D) or hMCP-1 (E). Effect of TAK-779 on hCCR2-induced chemotaxis in the presence of 4 nM of hMCP-1 (F). Each point represents means of the triplicate from one assay.

In order to confirm whether rabbit CCR2 expressed in U-937 cells is functionally coupled to G_αi _family of G proteins, chemotaxis assays were conducted using both parental U-937 and U-937/rabbit CCR2 stable transfectants. Cells were incubated in the top chamber in the presence of increasing concentrations of ligand in the lower chamber. Parental U-937 failed to migrate to either human MCP-1 or mouse JE. In contrast, U-937/rabbit CCR2 stable transfectants migrated effectively to either human MCP-1 or mouse JE (Fig. 6A and 6B). The maximal migration was observed at roughly 4 nM of ligand and ranged from 20–40 % total input compared to 0.5–1 % of cells migrating in the absence of any ligand. A typical bell-shaped curve was observed in the chemotaxis response with either human MCP-1 or mouse JE. As expected, both parental and rabbit CCR2 stable cell lines migrated well to the CXCR4 ligand SDF-1α (Fig. 6C). The antagonist effect of TAK-779 was also examined against rabbit CCR2 in the presence of defined concentration of either mouse JE or hMCP-1 (4 nM). Preincubation of rabbit CCR2 expressing cells with TAK-779 effectively blocked migration of either mouse JE or hMCP-1 with an *IC*_50 _of 98 nM and 4 nM, respectively (Fig. 6D and 6E). As expected, TAK-779 inhibited MCP-1 dependent migration of U-937/hCCR2b stable cells with an *IC*_50 _of 3.8 nM (Fig. 6F)

## Conclusion

In summary, we have cloned the full-length rabbit CCR2 cDNA and proved that the receptor is functional by expressing rabbit CCR2 in U-937 cells. The sequence of rabbit CCR2 will provide additional insight into the molecular evolution of chemokine receptors in different species. More importantly, the data obtained on cross-species activity of the CCR2 antagonist will provide valuable tools to perform toxicology and efficacy studies in the rabbit model.

## Methods

### Materials

Recombinant human chemokines (MCP-1, -2, -3, -4, RANTES, MIP-1α, MIP-1β) were purchased from Pepro Tech (Rocky Hill, NJ). Mouse JE and human SDF-1α were obtained from R&D Systems (Minneapolis, MN). Radiolabeled ligands (^125^I-human MCP-1 and ^125^I-mouse JE) were purchased from Perkin Elmer (Boston, MA). RPMI 1640, bovine albumin serum (BSA) and 4-(2-hydroxyethyl)-1-piperazineethanesulfonic acid (HEPES) were purchased from Sigma (Gaithersburgh, MD). Polymeric chain reaction (PCR) and Taqman primers were purchased from Qiagen (Valencia, CA). The CCR5/CCR2 dual antagonist TAK-779 was synthesized as described elsewhere [14].

### Rabbit cDNA cloning

Total RNA was prepared from the spleen of New Zealand white rabbits by the Trizol method and the cDNA was transcribed by using SuperScript™ First-Strand Synthesis System for RT-PCR (Invitrogen, Carlson, CA). Then the cDNA was amplified by PCR by using conserved primers (primer 1: 5'-GTATTCATCTTTGGTTTTGTGGGCAACATG-3' and primer 2: 5'-CAAAGGTAACTGTCCTGGCTTTTAAAGCAA-3'). The resultant PCR fragment was sequenced and this information was used to design the rabbit CCR2 gene specific primers. Rabbit CCR2 gene specific primers were used to amplify two fragments of rabbit cDNA by 5'- and 3'- rapid amplification of cDNA ends (RACE) using SMART RACE cDNA Amplification Kit (Clonetech, Palo Alto, CA). The sequence information from 5'- and 3'- RACE products was further used to design two primers (5'-GGTTGCTGAGAAGCCTGACACGC-3', and 5'-CAGGTCTGTATTCTTCAACAAGCCCTCG-3') out of start and stop codons, and full-length rabbit CCR2 cDNA was cloned using PCR amplification. The final PCR product with corresponding size was cloned to PCRII-TAPO (Invitrogen) and sequenced.

### Tissue distribution

Rabbit cDNA from brain, heart, liver, lung, spleen and testis were quantitated for expression levels of CCR2 and glyceraldehyde-3-phosphate dehydrogenase (GAPDH) by Taqman method. TaqMan primers and probes were designed using the Primer Express Computer Program (Applied Biosystems, Foster City, CA). The forward and reverse primers and probe selected for rabbit CCR2 are: 5'-catgacacactgctgcatcaac-3', 5'-gagaggtagctccggaacttctc-3' and 5'-[6-FAM]-ccgtggtctacgccttcgtcgg-[TAMRA-6-FAM]-3'. The forward, reverse primers and probe selected for rabbit GAPDH are: 5'-ggatttggccgcattgg-3', 5'-caacatccactttgccagagttaa-3' and 5'-[6-FAM]-cgcctggtcaccagggctgct-[TAMRA-6-FAM]-3'. Amplification mixture contained a total volume of 10 μl with 6 μl of TaqMan universal PCR master mixture (300 nM forward primer, 300 nM reverse primer, and 900 nM probe) and 4 μl of diluted samples. Taqman amplification was conducted on a ABI PRISM 7900HT Sequence Detector System (Applied Biosystems, Foster City, CA) with the following thermal profile: 1 cycle each of 50°C for 2 minutes and 95°C for 10 minutes followed by 40 cycles each of 95°C for 15 seconds and 60°C for 1 minute. Data was analyzed using a built-in standard curve method.

### Generation of retrovirus infected rabbit CCR2 stable cell line

Rabbit cDNA described above was amplified by PCR with primer 11: 5'-ggaggcagatctcgaacaggcagctggcgg-3' and primer 12: 5'-ttattggaattccaagccgacggctgtcca-3' (underlined nucleotides are the cleavage sites of *Bgl *II and *EcoR *I, respectively). The resulted PCR fragment was digested with *Bgl *II and *EcoR *I, and then subcloned into the corresponding site of pMSCV_puro _retroviral expression vector (BD Biosciences, CA). Lipofectamine-mediated transfection was carried out in the AmphoPack 293 packaging cell line (BD Biosciences, CA) with a full-length cDNA for rabbit CCR2 in the retroviral expression vector pMSCV_puro_, containing a puromycin resistance selection marker. Thirty six hours after transfection, the pool of viral supernatant was recovered from the packaging cells, centrifuged, and polybreen was added to a final concentration of 8 μg/ml. The viral supernatant was then added to parental U-937 cells in a tissue culture treated flask and the infection was allowed to proceed for 7 hours. At this time, additional medium was added to the packaging cells to generate more virus. After 7 hours, the second pool of viral supernatant was prepared as the first, except with 4 μg/ml polybreen. U-937 cells were resuspended in this viral supernatant and the infection was allowed to proceed overnight. Then the U-937 cells were placed into fresh medium without any selection. After 18 hours, the medium was changed to selection medium containing 0.4 μg/ml puromycin. The pool of stably transfected clones was propagated for binding and chemotaxis assays.

### Equilibrium binding assays

Equilibrium binding assay was conducted in RPMI 1640 medium with 10 mM HEPES and 0.2% BSA in room temperature with constant shaking for 2 hours. Reaction mixture contained a total volume of 100 μl with various concentrations of ^125^I-labeled ligands (^125^I-human MCP-1 or ^125^I-mouse JE), U-937/rabbit CCR2 stable transfectants (10^5 ^cells /well for ^125^I-human MCP-1 and 10^4 ^cells /well for ^125^I-mouse JE) in the presence or absence of corresponding cold ligand. Nonspecific binding was determined by 100-fold excess of cold ligand. Reactions were stopped by separation using the glass fiber filter (Wallac Printed Filtermat A) on a Tomtec Harvester 96-2 (Hamden, CT). Filters were subsequently washed 5 times with wash buffer (10 mM HEPES, 500 mM NaCl, pH 7.4) to remove the unbound radioligand. Finally, bound radioligand was quantitated using a liquid scintillation counter (Wallac 1205 Betaplate, Perkin Elmer). Dissociation constant (*K*_*d*_) and maximum binding (*B*_*max*_) were calculated by non-linear regression with Graphpad Prism 4.01 (San Diego, CA).

### Competition binding assays

The assay was conducted under the identical conditions as saturation binding assay described above with the radiolabeled ligand concentration fixed (0.11 nM ^125^I-mouse JE) in the presence of various concentrations of unlabeled chemokines or TAK-779. Values of *IC*_50 _were calculated by fitting the competition curves with Graphpad Prism 4.01.

### Chemotaxis assays

Chemotaxis assay was performed as described elsewhere [18] with a few modifications. Parental U-937 or U-937/rabbit CCR2 stable transfectants were harvested, counted and resuspended at a final cell density of 5 × 10^6 ^/ml in chemotaxis buffer (RPMI 1640 medium, containing 0.3% BSA and 1 mM HEPES). Chemokines were diluted to 1000 ng/ml stock and serially diluted (1:3) in migration buffer in a Costar 96 well plate (Cat #3790, Costar, Corning NY). Thirty microliters of each chemokine dilution were transferred to the corresponding well in the bottom chamber of a ChemoTX 96 well migration plate (5 μM pore size; Neuroprobe, Gaithersburg, MD). The ChemoTX filter unit was assembled and 50 μl of cells were added to the top chamber and incubated for 2.5 hours. Plates were then removed and cells were aspirated off the filter top. The cells that had migrated to the bottom chamber were pulsed with 5 μl of Aqueous One Solution (Promega, Madison, WI) and incubated for an additional 30–60 minutes at 37°C. Plates were shaken gently to permit uniform mixing and read at 490 nm in a Spectra_max _Plus reader (Molecular Dynamics, Piscataway, NJ). Cell standard curves were incorporated into the bottom of each plate with cell number ranges from 0–150,000 cells/well. The results were expressed as percent cell migration versus chemokine concentration. For compound testing, TAK-779 was diluted in DMSO at various concentrations and added to both the top and bottom chambers and chemotaxis assay was performed in the presence of 4 nM of mouse JE as described above.

## Abbreviations

CCR2, CC chemokine receptor 2; MCP, monocyte chemoattactant protein; RANTES, regulated on activation-regulated chemokine; MIP, macrophage inflammatory protein; GAPDH, glyceraldehyde-3-phosphate dehydrogenase; RACE, rapid amplification of cDNA ends, HEPES, 4-(2-hydroxyethyl)-1-piperazineethanesulfonic acid; BSA, bovine serum albumin; PCR, polymerase chain reaction.

## Authors' contributions

DL designed experiments, oversaw research and wrote the paper. X-JY conducted the experiments. RJE conducted the experiments. ATP conducted the experiments. HW and EWS participated in sequence alignments. CH synthesized the compound. CV conceived the study, designed experiments, oversaw research and wrote the paper.
